# Perinatal manifestations of congenital disorders of glycosylation—A clue to early diagnosis

**DOI:** 10.3389/fgene.2022.1019283

**Published:** 2022-12-13

**Authors:** Milena Greczan, Dariusz Rokicki, Dorota Wesół-Kucharska, Magdalena Kaczor, Agata Rawiak, Aleksandra Jezela-Stanek

**Affiliations:** ^1^ Department of Pediatrics, Nutrition and Metabolic Diseases, Children’s Memorial Health Institute, Warsaw, Poland; ^2^ Department of Genetics and Clinical Immunology, National Institute of Tuberculosis and Lung Disease, Warsaw, Poland

**Keywords:** CDG (congenital disorders of glycosylation), glycosylation, effusion, dysmorphia, thrombocytopenia, hydrops fetalis

## Abstract

N-glycosylation defects—isolated or mixed with other glycosylation defects—are the most frequent congenital disorders of glycosylation and present mostly in childhood, with a specific combination of non-specific phenotypic features. The diagnosis, however, is often delayed. The aim of this study is to describe the perinatal phenotype of congenital disorders of N-glycosylation. We present an analysis of perinatal symptoms in a group of 24 one-center Polish patients with N-glycosylation defects—isolated or mixed. The paper expands the perinatal phenotype of CDGs and shows that some distinctive combinations of symptoms present in the perinatal period should raise a suspicion of CDGs in a differential diagnosis.

## 1 Introduction

Congenital disorders of glycosylation (CDGs) are a group of inborn monogenic errors of glycoprotein and glycolipid formation and/or assembly (Chang et al., 2018; Altassan et al., 2019). The group is numerous, heterogeneous, and is divided into four subgroups, based on the pathomechanism: congenital disorders of N-glycosylation, disorders of O-glycosylation, disorders of glycolipids and glycophosphatidylinositol anchor synthesis (GPI-CDG), and mixed disorders of glycosylation (Chang et al., 2018).

Disorders of N-glycosylation form the most prevalent CDG subgroup, and their pediatric phenotype is well described. The symptoms usually involve multiple organs, and the course of the disease may be severe, milder with relapsing episodes of exacerbations and/or slowly improving (Péanne et al., 2018; Altassan et al., 2019). The most common features of N-glycosylation are dysmorphia, like inverted nipples, upslanted palpebral fissures, and abnormal fat pads over the buttocks; cerebellar hypoplasia; hepatopathy; nephromegaly; proteinuria; hypertension; hypoalbuminemia; coagulation defects (both pro- and anticoagulating factor deficiencies); cytopenias; and third-space effusions (Malhotra et al., 2009). All these symptoms, as well as generalized edema, cardiomyopathy, or cutis laxa, may be present also in the neonate (van de Kamp et al., 2007; Marklová and Albahri. 2008; Malhotra et al., 2009; Funke et al., 2013). Prenatally CDGs may manifest as non-immune hydros fettles, cerebellar hypoplasia, skeletal deformities, polyhydramnios or Ballantyne syndrome (Morava et al., 2012; Altassan et al., 2019; Čechová et al., 2020). Current knowledge on the perinatal manifestation is, however, still scarce. The literature claims that diagnosing CDG on the basis of clinical data during the neonatal period is not easy and requires a high index of suspicion (Edwards et al., 2006; Thong et al., 2009). Indeed, as supported by our experience, the diagnosis is usually delayed and is rarely made in the neonatal period (Funke et al., 2013; Schulte Althoff. 2016).

The aim of this study is to broaden the knowledge about the perinatal symptoms of CDGs by analyzing a group of Polish CDG patients.

## 2 Materials and methods

We identified 35 CDG patients hospitalized in our Institute in years 1995–2022; out of this group, 33 patients had N-glycosylation or mixed glycosylation defects. The group of 24 CDG patients, for whom perinatal data were available, was further analyzed.

## 3 Results

The analyzed group consisted of 24 patients: 19 with isolated N-glycosylation defects, i.e., 15 patients with PMM2-CDG, 2 with ALG1-CDG, 1 patient with DPAGT1-CDG, and 1 patient with MPI-CDG; 4 patients with the dolichol synthesis defect SRD5A3-CDG; and 1 patient with the disorder of Golgi homeostasis ATP6AP1-CDG. The detailed clinical information about the patients is summarized in Supplementary Table S1 and Table 1.

**TABLE 1:** Clinical symptoms found in different CDG types.

Symptom	PMM2-CDG *n* = 15	MPI-CDG *n* = 1	SRD5A3-CDG *n* = 4	ATP6AP1-CDG *n* = 1	ALG1-CDG *n* = 2	DPAGT1-CDG *n* = 1	Total *n* = 24
**Prenatal symptoms**	in 6/14 pts; 1 pt no data	in 0 pts	in 1 pt; 3 pts no data	in 1 pt	in 1 pt	in 1 pt	in 10/20
Poor fetal movements	3	-	-	-	-	-	3
Hydrops fetalis	1	-	-	1	-	-	2
Pericardial effusion	2	-	-	-	-	-	2
Hydrocele testis	1	-	-	-	-	-	1
Impending asphyxia	1	-	-	-	-	-	1
Multi-VSD	-	-	1	-	-	-	1
Ventricular cysts	-	-	1	-	-	-	1
Shortening of the long bones	-	-	1	-	-	-	1
Polyhydramnios	-	-	-	-	1	-	1
Hypotrophy	-	-	-	-	-	1	1
Oligohydramnios	-	-	-	-	-	1	1
Suspicion of situs inversus	-	-	-	-	-	1	1
**Dysmorphia**	in 9/12 pts; 3 pts no data	in 0 pts	in 3 pts	in 1 pt	in 2 pts	in 1 pt	in 16/21
Inverted nipples	8	-	-	-	-	-	8
Wide-set nipples	3	-	-	-	1	-	4
Upslanted palpebral fissures	2	-	-	-	-	-	2
Gothic palate	1	-	-	-	1	-	2
Low-set ears	1	-	1	-	1	-	3
Large ears	1	-	-	-	-	-	1
Orange peel skin	1	-	-	-	-	-	1
Abnormal fat pads	3	-	-	-	-	-	3
Joint contractures	4	-	-	-	-	1	5
Cryptorchidism	3	-	-	-	-	1	4
Generalized edema	6	-	-	-	-	-	6
Thin nasal bridge	1	-	-	-	-	-	1
Hypotelorism	1	-	-	-	-	-	1
Barrel-shaped chest	1	-	-	-	-	-	1
Long fingers	1	-	-	-	-	-	1
Hypotrophy	-	-	1	-	-	1	2
Hypertelorism	-	-	1	-	1	-	2
Downslanted palpebral fissures	-	-	1	-	-	-	1
Micrognathia	-	-	1	-	1	-	2
Retrognathia	-	-	1	-	1	-	2
Not specified facial dysmorphia	2	-	3	1	-	-	6
Bird-like nose	-	-	-	-	1	-	1
Microcephaly	-	-	-	-	1	-	1
Cutis laxa	-	-	-	1	-	-	1
**Adaptive disturbances**	in 10 pts	in 0 pts	in 1 pt	in 0 pts	in 1 pt	in 1 pt	in 14/24
Hypoglycemia	5	-	1	-	-	-	6
Transient respiratory problems	9	-	-	-	1	1	11
RDS (X-ray)	2	-	-	-	1	-	3
**Neurologic symptoms**	in 12/12 pts; 3 pts no data	in 0 pts	in 4 pts	no data	in 2 pts	in 1 pt	in 19/20
Hypotonia	8	-	3		1	-	12
Hypertonia	3	-	1		1	1	6
Central hypertonia with peripheral hypotonia	1	-	-		-	-	1
Poor sucking	4	-	-		1	-	5
Poorly expressed neonatal reflexes	3	-	-		1	-	4
Dystonia	-	-	1		-	-	1
Absent tendon reflexes	1	-	-		-	-	1
Overexpressed tendon reflexes	-	-	-		-	1	1
**Ocular symptoms**	in 13/13 pts; 2 pts no data	in 0 pts	in 4 pts	in 0 pts	in 1 pt	in 1 pt	in 19/22
Nystagmus	4	-	3	-	1	-	8
Roving eye movements	6	-	-	-	-	-	6
Strabismus	3	-	-	-	-	-	3
Pale retina	7	-	-	-	-	-	7
Hypoplastic macula	1	-	-	-	-	-	1
Hypoplastic optic nerve	1	-	-	-	-	-	1
Retinal pigment clumping	-	-	1	-	-	-	1
Cataract	-	-	-	-	-	1	1
**Hematological symptoms**	in 10/10 pts; 5 pts no data	in 0 pts	in 1/1 pt; 3 pts no data	in 1 pt	in 1/1 pt; 1 pt no data	in 1 pt	in 14/15
Thrombocytopenia	6	-	-	-	-	-	7
Anemia	5	-	1	1	-	1	8
Neutropenia	1	-	-	-	1	-	1
Thrombocythemia	1 + 1*	-	-	-	-	-	1 + 1*
Hyperleukocytosis	4 + 2*	-	-	-	-	-	5 + 2*
Elevated INR	2	-	-	-	1	-	2
Elevated APTT	2	-	-	-	-	-	2
Protein S deficiency	4/4	n/a	-	n/a	-	n/a	4
Protein C deficiency	3/4	n/a	n/a	n/a	n/a	n/a	5
AT III deficiency	4/5	n/a	n/a	n/a	1	n/a	5
Bruises, petechiae	5	-	n/a	-	1	-	6
Bleeding from mucosas	2	-	-	-	1	-	2
**Gastrointestinal**	in 6/15 pts	in 0 pts	in 0 pts	in 0 pts	in 2/2 pts	in 1/1 pt	9/24 pts
Regurgitations	3	-	-	-	1	1	5
Food retention	2	-	-	-	-	1	3
No swallowing reflex	0	-	-	-	1	-	1
Nasogastric tube	4	-	-	-	1	1	6
Parenteral nutrition	3	-	-	-	1	1	5
**Cardiac**	in 12/15 pts	no data	in 1/2 pts; 2 pts n/a	no data	in 1/2 pts	no data	14/19 pts
Pericardial effusion	9		-		-		9
HCM	3		-		-		3
IVS thickening	6		-		-		6
PFO	6		1		1		8
PDA	2		-		-		2
PST	1		-		-		1
Multi-VSD	-		1		-		1
Repolarisation abnormalities	1		-		-		1
Sinus tachycardia	1		-		-		1
**CNS pathology**	in 10/10 pts	no data	no data	no data	in 0/2 pts	in 1/1 pt	11/13 pts
Cerebellar hypoplasia	5				-	-	5
Ventriculomegaly	2				-	-	2
IVH	6				-	1	7
Pineal cyst	1				-	-	1
Arachnoid cyst	1				-	-	1
**Biochemical**	10 pts	no data	in 1 pt; 3 pts no data	in 1 pt	in 2/2 pts	No data	14/14
Hypoalbuminemia	7		1	n/a	2		10
Hypertransaminasemia	3		-	1	-		4
Hypocholesterolemia	5		n/a	1	1		7
Proteinuria	7		-	n/a	1		8
**Endocrine** hypothyreosis	3	0	0	0	0	0	3

Pts, patients; multi-VSD, multi-ventricular septal defect; RDS, respiratory distress syndrome; INR, international normalized ratio; APTT, activated partial thromboplastin time; AT III, antithrombin III; HCM, hypertrophic cardiomyopathy; IVS, intraventricular septum; PFO, patent foramen ovale; PDA, patent ductus arteriosus; CNS, central nervous system; IVH, intraventricular hemorrhage.

Majority of patients were delivered on term (87,5%, i.e., 21/24–21 patients of 24 for whom these data are available), with normal birth body weight (79,1%; i.e., 19/24); the median body weight was 3,000 g (15–50 percentile for the term neonate, according to WHO growth charts; range 1,360–4,180 g), median head circumference 33 cm (15–50 percentile; range 31–35 cm), and median birth body length 54 cm (>97 percentile; range 49–56 cm). The Apgar score was abnormal in 71% of patients (15/21), with abnormal results ranging from four to eight points in the first minute to five to nine points in the 10th minute and the lowest score in DPAGT1-and one ALG1-CDG patient (4–5). The delivery by cesarean section prevailed (69%, 9/13) and was caused by lack of labor progress (4), abnormal fetus position (2), premature labor (1), twin pregnancy in the systemic lupus erythematosus mother (1), and impending asphyxia in one case. The pregnancies were complicated in 8/24 cases (mother’s: thrombocytopenia in the ninth month (1), severe anemia (2), massive lower limb edemas (1), hypothyroidism (1), gestational diabetes mellitus type 2 (1), and pregnancy-induced hypertension (1)).

Perinatal symptoms were present in 79% of patients (19/24).

Prenatal symptoms were described in 50% of patients (10/20) and encompassed: poor fetal movements (3), mild pericardial effusion (2), non-immune hydrops fetalis (2), polyhydramnios (1), oligohydramnios, hypotrophy and suspicion of situs inverses (one, in a DPAGT1-CDG patient), impending asphyxia (1) and a combination of multi-ventricular septal defect (mVSD), ventricular cysts, and shortening of the long bones in one SRD5A3-CDG patient.

In all patients, some symptoms of illness were seen in the first days of life. Dysmorphic features were found in 76% (16/21) and included: inverted nipples (8), wide-set nipples (4), generalized edema (6), big joint contractures (5), abnormal fat distribution (3), low-set ears (3), upslanted palpebral fissures (2), hypotrophy (2), hypertelorism (2), gothic palate (2), micrognathia (2), retrognathia (2), and single cases of downslanted palpebral fissures, bilateral cataracts, high forehead, small forehead, barrel-shaped chest, bird-like nose, and hypotelorism. In boys, urogenital malformations were found, i.e., cryptorchidism (4/16 boys), hydrocele testis (2/16 boys), micropenis (1/16 boys), and hypoplastic scrotum (1/16 boys).

Almost half of the group suffered from transient adaptive respiratory problems starting in the first minutes to hours of life (45%, 11/24), with six patients requiring nCPAP or mechanical ventilation for 1–43 days and five requiring oxygen supplementation at least for a few hours. The chest X-ray revealed features of RDS (Respiratory Distress Syndrome) in three patients, including two prematurely born (29 and 32 weeks of gestation) and pneumonia in one. Six patients also had hypoglycemia in the first 1–2 days of life and received intravenous glucose infusions. Four patients developed jaundice, requiring phototherapy.

The neurological evaluation revealed muscle tone disturbances in 90% of patients (18/20), specifically 12 patients had hypotonia, while seven had hypertonia, which was combined with dystonia in one patient. In all patients, for whom those data were available (4/4), tendon reflexes were present in the neonatal period; however, in one, they were overexpressed. Neonatal reflexes were poorly expressed in seven patients, including poor sucking in four.

Another distinctive group of abnormalities found in the study group was ocular symptoms: nystagmus (64% of patients, 14/22), most often described as roving eye movements (6) or vertical–upward–eye movements (5). Horizontal nystagmus was found only in two patients. Three patients also had strabismus. In some patients, those symptoms were paroxysmal. Another prevalent ocular finding was pale retina (78%, 7/9), in four cases described even as “albinotic”. There were also single cases of retinal pigment clumping around the optic nerve, hypoplastic macula, oval optic disc, and bilateral cataracts.

Signs of coagulopathy such as bruises, petechiae, bleeding from injection sites, or bleeding from the mucosas were found in 29% of patients (7/24).

Gastrointestinal disturbances were present in 37% of patients (9/24): regurgitations, vomiting, poor sucking, and lack of swallowing reflex. Six patients required feeding by the nasogastric tube and/or parenteral nutrition (4).

In cardiological evaluation, the most common symptom was mild pericardial effusion, found in nine patients, and patent foramen ovale, (PFO) in eight. Mild cardiomyopathy was found in five patients, and isolated thickening of the intraventricular septum (IVS), in another five. Two patients suffered from cardiac arrhythmias from the first days of life, and it was paroxysmal supraventricular tachycardia (PST) in one and significant sinus tachycardia in the other. None of the patients required pericardial drainage in the neonatal period.

Brain imaging revealed intraventricular hemorrhage (IVH) I^o^ or II^o^ in 72% (6/8) of patients and ventriculomegaly (2/8) in ultrasound, while MRI scan revealed cerebellar hypoplasia in 72% (6/8) of examined patients.

In laboratory tests, hematological disturbances were prevalent, which concerned 78% of patients (14/18). The most frequent were cytopenia, i.e., severe thrombocytopenia (7) (transient in one case), thrombocytosis (1) (and one in the fourth week of life), anemia (7), hyperleukocytosis (5) (and one in the fourth week of life), and leukopenia (1). Coagulation assessment revealed profound antithrombin III deficiency in 83% (5/6) of patients, protein C in 83% (5/6), and protein S in 100% (4/4) examined patients; partial antithrombin time was elongated in two of three cases, and the international normalized ratio was slightly elevated in three of four patients (range 1,1–1,78). Biochemical tests revealed normal transaminases in 67% (8/12), while in the other four patients they were only mildly elevated; hypoalbuminemia was present in 100% of diagnosed patients (10/10), proteinuria in 10, and erythrocyturia in 4 patients. Also, thyroid gland evaluation was normal in 72% (8/11) of patients, with hypothyroidism found in the remaining three.

The course of the disease in the perinatal period was mild in 11 patients; thus, they were discharged in the first week of life. Patients more severely ill (14) required hospitalization for multiple weeks. No patient demised in the neonatal period. In all patients with severe course, some distinctive symptoms were more frequent, like generalized edema, hematological disturbances (thrombocytopenia or thrombocytosis, anemia, and hyperleukocytosis), and signs of coagulopathy, respiratory failure, pericardial effusion, and intolerance of enteral feeding.

None of our patients received a proper diagnosis in their neonatal period, and the median time to recognize the disease by transferrin isofocusing was six months of age (range 1–108 months). The most frequently noted initial diagnoses were congenital CMV infection, sepsis, RDS, myocarditis, pericarditis, pylorostenosis, cerebral palsy, and Smith–Lemli–Opitz syndrome.

## 4 Discussion

The group of congenital disorders of N-glycosylation consists of at least 31 diseases, with different enzyme or transporter deficiencies, which lead to a similar pathophysiological effect: defect of synthesis and/or processing of glycoproteins and glycolipids (Abu Bakar et al., 2018). Some types of CDGs have their distinctive features, but there is still a pool of symptoms that are common for a majority of N-glycosylation CDGs and some of the mixed glycosylation disorders involving the N-glycosylation pathway, like psychomotor retardation, hypotonia, peripheral neuropathy, stroke-like episodes, nephrotic syndrome, failure to thrive, nystagmus, strabismus, retinitis pigmentosa, third space effusions, cardiomyopathy, liver disease, or microcystic kidneys presenting as hyperechogenic in ultrasonography (USG) (Hertz-Pannier et al., 2006; Goreta et al., 2012; Öncül et al., 2022). Dysmorphic features usually described in those patients are inverted, wide-set nipples, abnormal fat pads, orange peel skin, high forehead, large ears, or thin upper lip (Verheijen et al., 2020). CDG patients with defective N-glycosylation also share common laboratory deviations such as hypertransaminasemia (most prominent in MPI-CDG, but also in others), hypoalbuminemia, low plasma cholesterol, laboratory indices of hypothyroidism (the actual cause of which is often a deficiency of the thyroxin-binding globulin), cytopenias, proteinuria, and serum clotting factor deficiencies (such as protein S, protein C, AT III, and factors IX, XI, II, V, VII, VIII, and X) (Truin et al., 2008; Verheijen et al., 2020).

N-glycosylation deficiency symptoms, such as non-immune hydrops fetalis, polyhydramnios, cardiomyopathy, pericardial effusions, skeletal abnormalities, or mirror syndrome (Ballantyne syndrome), may also occur prenatally (van de Kamp et al., 2007; Malhotra et al., 2009; Lei et al., 2021).

The results of our study confirm that many of aforementioned symptoms are present also in the perinatal period. In the analyzed group, we found pre- and neonatal non-immune generalized edemas, cardiomyopathy, pericardial effusions, and dysmorphic features like inverted, wide-set nipples, cryptorchidism, and abnormal fat pads, although according to the literature, the latter are rarely present in neonates (Goreta et al., 2012). In the first days of life, our patients also presented other typical symptoms, such as hypotonia, strabismus, failure to thrive, regurgitations, cerebellar hypoplasia, bleeding, coagulopathy, cytopenia (mainly thrombocytopenia or anemia), proteinuria, hypoalbuminemia, or serum clotting factor deficiencies.

Furthermore, our study also revealed some symptoms that are rarely described in the literature but were frequent in our study group and are discussed in the following paragraphs.

The interesting frequent finding (found in 45% of patients) is transient respiratory deterioration in the first minutes or hours of life, sometimes correlated with RDS features in RTG (mainly in prematurely born, but not only), requiring at least oxygen therapy or artificial ventilation for some time (range 1–43 days in the described group), and mostly not correlated with elevated inflammatory parameters. Such symptoms have been described in single cases previously, but its pathomechanism remains unknown (Schulte Althoff et al., 2016; van de Kamp et al., 2007). Hypothetically, pulmonary edema—as a part of generalized edema—might be the cause; another possibility is surfactant dysfunction due to hypoglycosylation of surfactant protein B and surfactant protein A (Taponen et al., 2013; Yang et al., 2014), but these considerations are beyond the subject of this paper.

Another finding rarely described previously is muscle hypertonia, which was present in seven patients, and a presence of tendon reflexes in all examined patients. According to the literature and personal experience, hyporeflexia is found in a majority of infants and older children with CDGs (Marklová and Albahri. 2007). Poor neonatal reflexes, including poor sucking and swallowing, were also frequent findings in the study group. This, together with enteral nutrition intolerance, manifested by typical regurgitations, vomiting, and undigested food retention, led to parenteral nutrition combined with nasogastric tube feeding in the neonatal period in a big fraction of our patients.

Not a common finding in the literature concerning CDGs, was also big joint contractures, found in 23% of patients in our study group.

An unusual type of nystagmus was prevalent in our cohort; it was described as roving eye movements or upward eye deviations and was often confused with epileptic seizures, particularly because it was paroxysmal in some patients. Another prevalent ocular finding was pale, albinotic retina, while in the literature, retinitis pigmentosa is described as the most frequent symptom (Lettice et al., 2010).

In addition to typical CDG hematological deviations, a part of our group presented transient significant hyperleukocytosis (30,144–80 × 10^9/L) that started in the neonatal period and normalized during the next few weeks of life and was not correlated with other signs or indices of infection. In two cases, it was also accompanied by significant thrombocytosis. According to our knowledge, such a phenomenon has never been described previously.

Interestingly, some of the well-known CDG symptoms have not been noted in a majority of the study group in their perinatal age, which are hyporeflexia, hypertransaminasemia, and hypothyroidism. Abdominal ultrasound was also normal in the majority of patients in their first days of life, without typical hepatomegaly or renal ultrasound findings, such as kidney enlargement, hyperechogenicity, and altered corticomedullary differentiation (Tiwary et al., 2022). Renal ultrasound abnormalities appeared gradually in the first few weeks or months of life in the described group.

More than half of our patients presented with a severe phenotype, which was more specific and easier to recognize. Still, almost the same number of patients showed a mild neonatal phenotype, and they were discharged from the hospital in the first weeks of life, without even posing a suspicion of CDGs. One of those patients with a “mild” neonatal phenotype (No. 5) presented to our hospital at the age of three months, in order to perform diagnostics of poor body weight gain. His/her dysmorphic features were highly suggestive of CDG, so during examination, the echocardiogram was performed, revealing impending cardiac tamponade. Another patient (No. 3), presented as a neonate with intensive vomiting and food retention and was suspected of having pylorostenosis. He/she underwent pyloroplasty in the third week of life, which was complicated by dehiscence of the stomach wall and general deterioration, partially due to *Pseudomonas aeruginosa* and *Candida albicans* sepsis. In addition, this surgery did not fix the enteral feeding problem, the patient required parenteral nutrition, so venesection was performed, which was complicated by the upper limb vein thrombosis.

This paper presents and extends the perinatal phenotype of congenital N-glycosylation disorders. It is important to emphasize that although the individual CDG symptoms seem to be non-specific, the occurrence of a combination of several of them, with the involvement of different systems, should direct the differential diagnosis specifically toward congenital disorders of N-glycosylation. Despite the specific treatment being available only to a few of them, early diagnosis may influence patient’s further medical care and genetic counseling for the family, concerning family planning. It may reduce a number of procedures, which are at higher risk in this group of patients, such as surgery, and monitor patients for the occurrence of potential life-threatening complications of the disease.

## Data Availability

The original contributions presented in the study are included in the article/Supplementary Materials, further inquiries can be directed to the corresponding author.

## References

[B1] Abu Bakar N. Lefeber D. J. van Scherpenzeel M. (2018). Clinical glycomics for the diagnosis of congenital disorders of glycosylation. J. Inherit. Metab. Dis. 41 (3), 499–513. 10.1007/s10545-018-0144-9 29497882 PMC5959975

[B2] Altassan R. Péanne R. Jaeken J. Barone R. Bidet M. Borgel D. (2019). International clinical guidelines for the management of phosphomannomutase 2-congenital disorders of glycosylation: Diagnosis, treatment and follow upErratum in. J. Inherit. Metab. DisJ Inherit. Metab. Dis. 4242 (13), 5577–5628. PMID: 30740725. 10.1002/jimd.12024 30740725

[B3] Čechová A. Altassan R. Borgel D. Bruneel A. Correia J. Girard M. (2020). Consensus guideline for the diagnosis and management of mannose phosphate isomerase-congenital disorder of glycosylation. J. Inherit. Metab. Dis. 43 (4), 671–693. 10.1002/jimd.12241 32266963 PMC7574589

[B4] Chang I. J. He M. Lam C. T. (2018). Congenital disorders of glycosylation. Ann. Transl. Med. 6 (24), 477. PMID:30740408; PMCID: PMC6331365. 10.21037/atm.2018.10.45 30740408 PMC6331365

[B5] Edwards M. McKenzie F. O'callaghan S. Somerset D. Woodford P. Spilsbury J. (2006). Prenatal diagnosis of congenital disorder of glycosylation type Ia (CDG-Ia) by cordocentesis and transferrin isoelectric focussing of serum of a 27-week fetus with non-immune hydrops. Prenat. Diagn. 26, 985–988. 10.1002/pd.1543 16915591

[B6] Funke S. Gardeitchik T. Kouwenberg D. Mohamed M. Wortmann S. B. Korsch E. (2013). Perinatal and early infantile symptoms in congenital disorders of glycosylation. Am. J. Med. Genet. A 3, 578–584. 10.1002/ajmg.a.35702 23401092

[B7] Goreta S. S. Dabelic S. Dumic J. (2012). Insights into complexity of congenital disorders of glycosylation. Biochem. Med. 22 (2), 156–170. 10.11613/bm.2012.019 PMC4062342 22838182

[B8] Hertz-Pannier L. Dechaux M. Sinico M. Emond S. Cormier-Daire V. Saudubray J. M. (2006). Congenital disorders of glycosylation type I: A rare but new cause of hyperechoic kidneys in infants and children due to early microcystic changes. Pediatr. Radiol. 36, 2108–2114. 10.1007/s00247-005-0001-5 16328327

[B9] Lei Y-L. Zhen L. Xu L. L. Yang Y. D. Li D. Z. (2021). Foetal phenotype of ALG1-CDG caused by paternal uniparental disomy 16. J. Obstet. Gynaecol. 41 (5), 828–830. 10.1080/01443615.2020.1786031 32811240

[B10] Léticée N. Bessières-Grattagliano B. Dupré T. Vuillaumier-Barrot S. de Lonlay P. Razavi F. (2010). Should PMM2-deficiency (CDG Ia) be searched in every case of unexplained hydrops fetalis? Mol. Genet. Metab. 101 (2-3), 253–257. Epub 2010 Jun 22. PMID: 20638314. 10.1016/j.ymgme.2010.06.009 20638314

[B11] Malhotra A. PatemAn A. Coman D. Menahem S. (2009). Prenatal cardiac ultrasound finding in congenital disorder of glycosylation type 1a. Fetal diagn. Ther. 251, 54–57. 10.1159/000196816 19176971

[B12] Marklová E. Albahri Z. (2007). Screening and diagnosis of congenital disorders of glycosylation. Clin. Chim. Acta. 385 (1-2), 6–20. Epub 2007 Jul 13. PMID: 17716641. 10.1016/j.cca.2007.07.002 17716641

[B13] Morava E. Vodopiutz J. Lefeber D. J. Janecke A. R. Schmidt W. M. Lechner S. (2012). Defining the phenotype in congenital disorder of glycosylation due to ALG1 mutations. Pediatrics 130, e1034–e1039. 10.1542/peds.2011-2711 22966035

[B14] Öncül U. Kose E. Eminoğlu F. T. (2022). ALG1-CDG: A patient with a mild phenotype and literature review. Mol. Syndromol. 13 (1), 69–74. Epub 2021 Sep 21. PMID: 35221878; PMCID: PMC8832214. 10.1159/000517797 35221878 PMC8832214

[B15] Péanne R. de Lonlay P. Foulquier F. Kornak U. Lefeber D. J. Morava E. (2018). Congenital disorders of glycosylation (CDG): Quo vadis? Eur. J. Med. Genet. 61, 11184–11663. 10.1016/j.ejmg.2017.10.012 29079546

[B16] Schulte Althoff S. GrunebergM. Reunert J. Park J. H. RuSt S. Muhlhausen C. (2016). TMEM165 deficiency: Postnatal changes in glycosylation. JIMD Rep. 26, 21–29. 10.1007/8904_2015_455 26238249 PMC5580733

[B17] Taponen S. Huusko J. M. Petaja-Repo U. E. Paananen R. Guttentag S. H. Hallman M. (2013). Allele-specific N-glycosylation delays human surfactant protein B secretion *in vitro* and associates with decreased protein levels *in vivo* . Pediatr. Res. 74 (6), 646–651. 10.1038/pr.2013.151 24002332

[B18] Thong M. K. FietzM. NiCholls C. Lee M. H. Asma O. (2009). Congenital disorder of glycosylation type ia in a Malaysian family: Clinical outcome and description of a novel PMM2 mutation. J. Inherit. Metab. Dis. 32, S41–S44. 10.1007/s10545-009-1031-1 19165618

[B19] Tiwary H. Hecht L. E. Brucker W. J. Berry G. T. Rodig N. M. (2022). The development of end stage renal disease in two patients with PMM2-CDG. JIMD Rep. 63 (2), 131–136. PMCID: PMC8898725. PMID: 35281664. 10.1002/jmd2.12269 35281664 PMC8898725

[B20] Truin G. Guillard M. Lefeber D. J. Sykut-Cegielska J. Adamowicz M. Hoppenreijs E. (2008). Pericardial and abdominal fluid accumulation in congenital disorder of glycosylation type Ia. Mol. Genet. Metab. 94 (4), 481–484. 10.1016/j.ymgme.2008.05.005 18571450

[B21] van de Kamp J. M. Ruijter G. J. G. Steggerda S. J. den Hollander N. S. Willems S. M. Matthijis G. (2007). Congenital disorder of glycosylation type Ia presenting with hydrops fetalis. J. Med. Genet. 44 (4), 277–280. 10.1136/jmg.2006.044735 17158594 PMC2598051

[B22] Verheijen J. Tahata S. Kozicz T. Witters P. Morava E. (2020). Therapeutic approaches in congenital disorders of glycosylation (CDG) involving N-linked glycosylation: An update. Genet. Med. 22 (2), 268–279. 10.1038/s41436-019-0647-2 31534212 PMC8720509

[B23] Yang W. Shen H. Fang G. Li H. Li L. Deng F. (2014). Mutations of rat surfactant protein A have distinct effects on its glycosylation, secretion, aggregation and degradation. Life Sci. 117 (2), 47–55. 10.1016/j.lfs.2014.09.006 25242514

