# Supplementary material for: Perinatal manifestations of congenital disorders of glycosylation—A clue to early diagnosis
Source: Front Genet. 2022 Dec 13;13:1019283. doi: 10.3389/fgene.2022.1019283 (PMC9792486; doi:10.3389/fgene.2022.1019283)
Supplement: Supplementary file 1 [file Table1.pdf]

Table 1. Clinical data of described patients

No	Disease	Sex	Birth data	Prenatal sympto-ms	Dysmorphia	Adaptive disturban-ces	Neurologi-cal	Ocular	Hematological	Gastroin-testinal	Cardiac patholo-gy	CNS pathology	Biochemical laboratory tests	Abdomi-nal USG	IEF at the age of (months):
1	**PMM2-CDG**<br>c.155T>G, p.Val52Gly; c.640-23A>G, p.?	M	Hbd 37<br>W 2860g<br>L 54 cm<br>HC 34 cm<br>Apgar 10	no	no	Hypoglycemia	n/a	Nystagmus	AT III 97,8%<br>Protein C 113,6%<br>Protein S 48,1%	no	no	MRI:<br>- cerebellar hypoplasia<br>- pineal cyst	AST/ALT normal<br>Albumin n/a<br>T Chol 140 mg/d<br>TSH normal<br>No proteinuria	normal	60
2	**PMM2-CDG**<br>c.710C>G, p.Thr237Arg; c.691G>A, p.Val231Met	F	Hbd 39<br>W 3100g<br>L n/a<br>HC n/a<br>Apgar 7/9	Poor fetal movements	Inverted nipples<br>Wide set nipples<br>Upslanted palpebral fissures<br>Gothic palate<br>Low set ears<br>Orange peel skin on arms, thighs and buttocks<br>Abnormal fat pads over the buttocks<br>Knees and hips contractures	Transient respiratory disturbances<br>Required use of Ambu bag and nCPAP for 5 min<br>Tachypnea	Muscle hypotonia<br>Poorly expressed neonatal reflexes<br>No sucking reflex<br>Tendon reflexes present	Nystagmus, roving eye movements<br>Pale, “albinotic” retina<br>Hypoplastc macula	Bruises<br>Bleeding from the injection sites and mucosa<br>APTT 49-73 s<br>INR 1,14-1,63<br>Severe thrombocytopenia<br>Anemia<br>HyperleukocytosIs without infection (peak of 29x10^9/ul in 8 day of life)	Poor sucking reflex<br>Feeding by nasogastric tube + perenteral nutrition	PFO<br>IVS thickening	USG:<br>Ventriculomega-ly, IVH I<br>MRI:<br>-cerebellar hypoplasia	AST/ALT normal<br>Hypoalbuminemia<br>T Chol 82mg/dl<br>Hypothyreosis<br>Proteinuria	Mild dilatation of renal pyelocalices	2

3	**PMM2-CDG**<br><br>c.640-15479 C>T (deep intronic splice mutation); c.691G>A, p.Val231Met	M	Hbd 38<br>W.4180 g<br>L n/a<br>HC n/a<br>Apgar 8	Poor fetal movements	Unilateral cryptorchidism<br><br>Inverted nipples<br><br>Generalized oedema<br><br>Hip contractures	Grunting<br><br>Peripheral cyanosis<br><br>Dyspnea<br><br>Respiratory insufficiency - required oxygen<br><br>Arrythmia (PST)<br><br>Deterioration in the 1st day of life - mechanical ventilation<br><br>Hypoglycemia	Muscle hypotonia<br><br>Tendon reflexes present<br><br>Poorly expressed neonatal reflexes	Horizontal nystagmus	Facial petechiae<br>Erythrocyturia<br><br>APTT, INR normal<br>AT III decreased<br><br>Mild thrombocytopenia<br>Transient hyperleukocytosis 30 kk/ul	Regurgitations, vomiting<br><br>Parenteral nutriton	PFO<br><br>Pericardial effusion 3 mm<br><br>RV and IVS hypertro-phy<br><br>PST 2x	<u>USG:</u> normal	AST/ALT n/a<br><br>Hypoalbuminemia<br><br>T Chol 83 mg/dl<br><br>TSH normal<br><br>No proteinuria	Hyperechogenic and enlarged kidneys	4
4	**PMM2-CDG**<br>c.422G>C, p.Arg141His; c.691G>A, p.Val231Met	M	Hbd 40<br>W 3770 g<br>L n/a<br>HC n/a<br>Apgar 9	Hydrops fetalis<br><br>Pericardial effusion 7 mm<br><br>Hydrocele testis	Inverted nipples<br><br>Not specified facial dysmorphia<br><br>Generalized oedema	Respiratory disturbances - required oxygen<br><br><u>X-ray:</u><br>RDS I	Muscle hypertonia<br><br>Poor suckling reflex	Nystagmus - roving eye movements mainly upwards<br><br>Strabismus	Protein C 10,9%<br>Protein S 30,8%<br><br>*Transient hyperleukocytosis Thrombocythemia in 4th week of life without infection	no	PDA<br><br>Pericardial effusion 7 mm<br><br>Thickening of the myocardium<br><br>Repolarisa-tion abdnormali-ties	<u>MRI:</u><br>-cerebellar hypoplasia	AST/ALT normal<br><br>Hypoalbuminemia<br><br>T Chol 86 mg/dl<br><br>TSH normal<br><br>Proteinuria	Normal	6
5	**PMM2-CDG**<br>c.422G>C, p.Arg141His; c.691G>A, p.Val231Met	F	Hbd 40<br>W 2880 g<br>L n/a<br>HC n/a<br>Apgar 10	Pericardial effusion 3 mm	Inverted nipples<br><br>Wide set nipples<br><br>Fat pads over the buttocks	no	Muscle hypotonia<br><br>Poor sucking reflex	Episodes of nystagmus - roving eye movements<br><br>Strabismus	n/a	no	Pericardial effusion 5 mm	n/a	n/a	n/a	3

								Pale, translucent, “albinotic” retina							
**6**	**PMM2-CDG**<br>c.422G>C**,**<br>p.Arg141His;<br>c.691G>A,<br>p.Val231Met	M	Hbd<br>“term”<br>W 3100 g<br>L n/a<br>HC n/a<br>Apgar n/a	Poor fetal movements	Thin nasal bridge<br><br>Hypotelorism<br>Ear deformation<br><br>Barrel-shaped chest<br><br>Long fingers	no	Muscle hypotonia	Pale, “albinotic” retina	Anemia<br>Transient thrombocythemia<br>734x10^3/ul<br><br>INR 1.1	no	Mild pericardial effusion	n/a	AST 199 U/l<br>ALT 94 U/l<br>albumin n/a	n/a	7
**7**	**PMM2-CDG**<br>c.422G>C,<br>p.Arg141His;<br>c.722G>C,<br>p.Cys241Ser	F	Hbd<br>“term”<br>W 3725 g<br>L 56 cm<br>HC 35 cm<br>Apgar 10	no	n/a	no	Muscle hypotonia	n/a	lab n/a	n/a	n/a	n/a	n/a	n/a	48
**8**	**PMM2-CDG**<br>c.24delC,<br>p.C9AfsX27;<br>c.691G>A,<br>p.Val231Met	F	Hbd 41<br>W 2700 g<br>L n/a<br>HC n/a<br>Apgar 7/8	no	n/a	Grunting, dyspnea<br><br>Respiratory insufficiency - required oxygen, nCPAP and ventilation<br><br>X-ray: pneumonia	Muscle hypertonia	Nystagmus: horizontal and vertical – upwards eye deviations<br><br>Pale retina<br><br>Hypoplas-tic optic disc	Bleeding from respiratory and gastrointestinal mucosa<br><br>Severe anemia (Hb 6.4 g/dl)<br>Severe thrombocytopenia<br>Hyperleucytosis (34x10^9/ul)<br><br>AT III 13,52%<br>Protein C 6%<br>Protein S 38%	Vomiting, food retention - nasogastric tube + parenteral nutrition	PFO<br><br>IVS thickening<br><br>Sinus tachycardia	USG:<br>IVH II<br><br>MRI:<br>cerebellar hypoplasia	AST 131 U/l<br>ALT 92 U/l<br><br>Albumin n/a<br><br>Hypothyreosis<br><br>Proteinuria	Hyperechoge-nic kidneys<br><br>Dilated pyelocalices	1
**9**	**PMM2-CDG**<br>c.422G>C,<br>p.Arg141His;<br>c.691G>A,	M	Hbd 39<br>W 3900 g<br>L n/a<br>HC n/a<br>Apgar 10	n/a	Inverted nipples<br><br>Wide set nipples	Termoregulati on disturbances	Central hypertonia and peripheral hypotonia	Nystagmus - roving eye movements	Bruises<br>Erythrocyturia<br><br>APTT 84,2 s.<br>INR 1,78	Regurgitati on, choking - nasogastric	PFO<br><br>Pericardial effusion mild	USG:<br>IVH I<br><br>MRI:	AST 75 U/l<br>ALT 38 U/l<br><br>Hypoalbuminemia	Normal	1,5

	p.Val231Met				Cryptorchidi-sm<br><br>Upslanted palpebral fissures<br><br>Generalized oedema	Tachypnea, required oxygen for 1 day<br><br>Hypoglycemia	Absent tendon reflexes<br><br>Poorly expressed neonatal reflexes	Episodes of strabismus<br><br>Pale, translucent retina	ATIII 25%<br>Protein C 13,2%<br>Protein S 42,7%<br><br>Severe thrombocytopenia	tube + oral feeding	Thickening of IVS and RV	wide subarachnoid spaces	TSH normal<br><br>Proteinuria		
**10**	**PMM2-CDG**<br><br>n/a	M	Hbd 40<br>W 4100 g<br>L n/a<br>HC n/a<br>Apgar n/a	no	Inverted nipples<br><br>Large ears	no/a	n/a	Vertical nystagmus – upwards eye deviations	lab n/a	no	Mild pericardial effusion<br><br>IVS thickened	n/a	n/a	n/a	5
**11**	**PMM2-CDG**<br><br>n/a	F	Hbd 34<br>W 2810 g<br>L 51 cm<br>HC 32 cm<br>Apgar 10	no	Generalized oedema<br><br>Not specified facial dysmorphia	Grunting<br><br>Required oxygen<br><br>Hypoglycemia	Poor sucking reflex	Nystagmus - roving eye movements , mainly upwards<br><br>Pale retina<br><br>Oval optic disc	Transient hyperleukocytosis (37 x 10^9/ul) lasting for few weeks (with a peak of 80 10^9/ul) without infection<br>Anemia<br><br>AT III 25,1%	Nasogastric tube from 5th day of life	PFO<br><br>IVS thickening	USG:<br>IVH I	AST 26 U/l<br>ALT 13 U/l<br><br>Hypoalbuminemia<br><br>T Chol 70 mg/d<br><br>TSH norma<br><br>Proteinuria	Normal	2
**12**	**PMM2-CDG**<br>c.484C>T, p.Arg162Trp; c.422G>C, p.Arg141His	M	Hbd 40<br>W 3200 g<br>L 55<br>HC 33<br>Apgar 6/8	no	n/a	Respiratory insufficiency - required oxygen	Muscle hypotonia	n/a	n/a	no	no	USG:<br>IVH I	n/a	n/a	108
**13**	**PMM2-CDG**<br><br>n/a	M	Hbd 32<br>W 2050 g<br>L n/a<br>HC 33 cm<br>Apgar 8/9	Impending asphyxia	Inverted nipples<br><br>Generalized oedema<br><br>Long head	Respiratory insufficiency, required oxygen<br><br>X-ray: RDS	Muscle hypertonia	Pale, “albinotic” retina	Petechiae<br>Erythrocyturia<br>Transient hyperleukocytosis (peak of 70 x 10^9/ul from 2nd week of life to 4th week of life) without infection	Food retention	PFO, PDA<br><br>Pericardial effusion<br><br>Hypertrophic cardiomyopat hy	USG:<br>IVH II<br><br>arachnoid cyst<br><br>ventriculome galy	AST/ALT normal<br><br>Hypoalbuminemia<br><br>TSH normal<br><br>Proteinuria	Normal	2

					Big joints contractures				Severe thrombocytopenia<br>Anemia						
**14**	**PMM2-CDG**<br>c.24delC, p.C9AfsX27; c.385G>A, p.Val129Met	M	Hbd 38<br>W 3730 g<br>L 59 cm<br>HC n/a<br>Apgar 5/8	no	Inverted nipples<br><br>Generalized oedema<br><br>Umbilical cord swelling<br><br>Fat pads over the buttocks<br><br>Hypoplastic scrotum<br><br>Cryptorchi-dism<br><br>Wrists contractures	Respiratory failure, required mechnical ventilation in the 1st day of life<br>Hypoglycemia	Muscle hypotonia	Nystagmus - roving eye movements upwards eye deviations	Bruises<br><br>Episodes of coagulopathy<br><br>Leukopenia<br><br>Severe thrombocytopenia	no	Pericardial effusion 5 mm	MRI: cerebellar hypoplasia	AST 100 U/l<br>ALT 76 U/l<br><br>Hypoalbuminemia<br><br>T Chol 79 mg/dl<br><br>Hypothyreosis<br><br>Proteinuria	n/a	7
**15**	**PMM2-CDG**<br><br>n/a	M	Hbd 40<br>W 2900 g<br>L n/a<br>HC n/a<br>Apgar 10	no	no	no	n/a	no	n/a	no	Mild pericardial effusion<br><br>Hypertrophic cardiomyopa-thy	n/a	n/a	n/a	6
**16**	**MPI-CDG**<br>c.656G>A, p.Arg129Gln; c.748G>A, p.Gly250Ser	F	Hbd 41<br>W 3300 g<br>L 59<br>HC n/a<br>Apgar 6/7	no	**no**	no	no	no	n/a	no	n/a	n/a	n/a	n/a	22
**17**	**SRD5A3-CDG**<br><br>c.292_293del, p.Leu98ValfsX121 homozygous	F	Hbd "term"<br>W 2900 g<br>L 49 cm<br>HC 33 cm	n/a	Not specified facial dysmorphia	no	Hypertonia<br><br>Dystonia	Nystagmus	n/a	no	n/a	n/a	n/a	n/a	n/a

			Apgar 8												
18	**SRD5A3-CDG**<br>c.292_293del,<br>p.Leu98ValfsX121<br>homozygous	F	Hbd<br>“term”<br>W 2490 g<br>L 50 cm<br>HC 33 cm<br>Apgar n/a	n/a	Not specified facial dysmorphia	no	Muscle hypotonia	Nystagmus	n/a	no	n/a	n/a	n/a	n/a	n/a
19	**SRD5A3-CDG**<br>c.424C>T,;<br>p.Arg142X<br>homozygous	M	Hbd 40<br>W 2560 g<br>L 49 cm<br>HC n/a<br>Apgar 7/8	mVSD<br><br>Ventricular cysts<br><br>Shortening of the long bones	Hypotrophy<br>Hypertelorism<br><br>Downslanted palpebral fissures<br><br>Prominent forehead<br><br>Micrognathia<br><br>Retrognathia<br><br>Low set ears	Hypoglycemia	Muscle hypotonia	Retinal pigment clumping around optic nerve	Transient thrombocytopenia in the first days of life	no	PFO<br>mVSD	n/a	AST/ALT normal<br><br>Hypoalbuminemia<br><br>TSH normal	Mild dilatation of the renal left pyelocalix	2
20	**SRD5A3-CDG**<br>c.489C>A,<br>p.Tyr163Ter;<br>c.424C>T,<br>p.Arg142X	M	Hbd<br>“term”<br>W 3300 g<br>L n/a<br>HC n/a<br>Apgar 10	n/a	no	no	Muscle hypotonia	Nystagmus	n/a	no	no	n/a	n/a	n/a	12
21	**ATP6AP1-CDG**<br>c.1284G>A,<br>p.Met428Ile<br>X-linked	M	Hbd 38<br>W 3600 g<br>L 54 cm<br>HC 34 cm<br>Apgar 8	Hydrops fetalis	Not specified dysmorphia	no	n/a	no	Anemia	no	n/a	n/a	AST 111 U/l<br>ALT 32 U/l<br><br>Albumin n/a<br><br>T Chol 64 mg/dl	no	n/a

**22**	**ALG1-CDG**<br>c.773C>T,<br>p.Ser258Leu;<br>c.1182C>G,<br>p.Phe394Leu	M	Hbd 29<br>W 1360 g<br>L n/a<br>HC n/a<br>Apgar 5	Polyhydra-<br>mnios	Bird-like nose<br><br>Hypertelorism<br><br>Retrognathia<br><br>Gothic palate<br><br>Low set ears<br><br>Wide set nipples	Respiratory failure<br><br>X-ray: RDS III/IV<br><br>Required surfactant and mechanical ventilation for 8 days	Muscle hypotonia<br><br>Poorly expressed neonatal reflexes<br><br>No sucking reflex	no	Subcutaneus hemorrhages<br>Erythrocyturia<br><br>APTT 36,5s<br>INR 1,33<br>ATIII 21,93%<br>Protein C 7,8%<br><br>Severe anemia	No sucking and swallowing reflex<br>-<br>nasogastric tube +<br>parenteral nutrition	PFO	<u>MRI:</u> normal	AST/ALT normal<br><br>Hypoalbuminemia<br><br>T Chol 59 mg/dl<br><br>TSH normal<br><br>Proteinuria	Mild dilatation of renal pyelocalices	18
**23**	**ALG1-CDG**<br>c.773C>T,<br>p.Ser258Leu;<br>c.1182C>G,<br>p.Phe394Leu	M	Hbd 37<br>W 2800 g<br>L n/a<br>HC 31 cm<br>Apgar 7/10	no	Microcephaly<br><br>Small forehead<br><br>Micrognathia	no	Muscle hypertonia	Nystagmus	n/a	Regurgita-<br>tions	no	<u>MRI:</u> normal	AST/ALT n/a<br><br>Hypoalbuminemia	n/a	3
**24**	**DPAGT1-CDG**<br>p.1117C>G,<br>p. Pro373Ala;<br>c.1197T>A,<br>p.Tyr399X	M	Hbd 37<br>W 1410 g<br>L n/a<br>HC n/a<br>Apgar 4/8<br>Second twin	Hypotrophy<br><br>Oligohydramn<br>ios<br><br>Suspicion of situs inversus	Severe hypotrophy<br><br>Cryptorchi-<br>dism<br><br>Elbow joint contractures<br><br>Bilateral cataracts	Respiratory disturbances, required oxygen and then nCPAP in 2nd day of life	Muscle hypertonia,<br><br>Overexpre-<br>ssed tendon reflexes	Bilateral cataract	Anemia	Food retention, regurgita-<br>tions<br><br>Parenteral nutrition until 24th day of life<br>Nasogastric tube	n/a	<u>USG:</u><br>IVH I/II	n/a	Normal	6

M – male, F – female, Hbd – week of pregnancy, W – weight, L – length, HC – head circumference, nCPAP – nasal Continuous Positive Airway Pressure, PST – paroxysmal supraventricular tachycardia, RDS – respiratory distress syndrome, AT III – antithrombin III, APTT - activated partial thromboplastin time, INR - International Normalized Ratio, PFO – patent foramen ovale, IVS – intraventricular septum, RV – right ventricle, PDA – patent ductus arteriosus, mVSD – multi-ventricular septal defect, USG – ultrasonography, IVH – intraventricular hemorrhage, MRI – magnetic resonance imaging, AST – aspartate transaminase, ALT – alanine transaminase, T chol – total cholesterol, TSH – thyroid stimulating hormone
